# Understanding patient preferences on providing sociodemographic information in an acute care setting: a qualitative study

**DOI:** 10.1186/s12913-025-13708-3

**Published:** 2025-11-22

**Authors:** Yasmin Garad, Negin Pak, Bronwyn Barker, Danielle Kasperavicius, Joshua Pratt, Seema Marwaha, Michael Colacci, Reena Pattani, Elizabeth Kipp, Joan Conrad, Sharon E. Straus, Christine Fahim

**Affiliations:** 1 Knowledge Translation Program, St. Michael’s Hospital, Unity Health Toronto, 209 Victoria St, Toronto, ON M5B 1T8 Canada; 2 Institute of Health Policy, Management and Evaluation, University of Toronto, 155 College St, Toronto, ON M5T 3M6 Canada; 3 Division of General Internal Medicine, Department of Medicine, St. Michael’s Hospital, Unity Health Toronto, 209 Victoria St, Toronto, ON M5B 1T8 Canada; 4 Department of Medicine, University of Toronto, 6 Queens Pk Cres W 3rd Floor, Toronto, ON M5S 3H2 Canada; 5 Caregiver partner, Ottawa, ON Canada; 6 Cancer Care Ontario, 620 University Ave, Toronto, ON M5G 2C1 Canada

**Keywords:** Health equity, Patient perspectives, Qualitative research, Patient privacy, Sociodemographic data, Routine data collection

## Abstract

**Background:**

Access to patient sociodemographic information is a critical factor to understanding, at a systems-level, where health inequities exist so they can be addressed. Patient concerns around disclosing personal information remain a barrier to sociodemographic data collection. Our objective was to assess patient perceptions regarding the routine collection of sociodemographic information in an acute care hospital setting.

**Methods:**

We conducted a qualitative study using the Framework Method to understand patient perceptions regarding sociodemographic data collection. We administered semi-structured interviews with patients admitted to the General Internal Medicine and Geriatric Medicine units at a university-affiliated hospital in Toronto, Canada. Two reviewers independently coded 10% of interview transcripts until a kappa ≥ 0.7 was achieved. The remaining interviews were single-coded. Data were analyzed using thematic analysis.

**Results:**

A total of 52 qualitative interviews were conducted between December 2021 and September 2023. Of the 52 individuals interviewed, 21 also agreed to complete a sociodemographic survey (40%). Among these participants, 57% (*n* = 12) were women and 62% (*n* = 13) were White. Patients felt more comfortable disclosing sociodemographic data if they believed it would lead to better or more equitable care; data were collected after they were admitted to hospital; data were collected verbally; and concerns about data privacy, anonymization, use, and secure storage were addressed. Some patients expressed discomfort being asked questions about income or race. Most patients had no preference regarding who from the healthcare team collected the sociodemographic data. Participants reported the importance of a friendly and respectful approach of the person collecting the data.

**Conclusions:**

Participants reported feeling comfortable disclosing their sociodemographic information in hospital; however, only 40% were willing to complete a demographic questionnaire. Participants’ comfort levels were impacted by the approach of the individual asking the questions, the types of questions asked, when the data were collected, and whether assurances around privacy and transparency regarding the use of data were provided. The results of this study should be used to develop strategies to support the implementation of routine sociodemographic data collection in acute care settings.

**Supplementary Information:**

The online version contains supplementary material available at 10.1186/s12913-025-13708-3.

## Background

Addressing health disparities is essential to promoting equitable access to healthcare, particularly for individuals from structurally marginalized communities. In Canada, inequities in health outcomes have been associated with many intersecting socioeconomic factors, including immigration, racialization, low income, insecure employment, limited educational attainment, and belonging to a sexual or gender minority [1–7]. Individuals affected by these factors typically face systemic challenges such as racism, discrimination, poor nutrition, disadvantaged living conditions and less access to healthcare resources, which in turn contributes to poorer health and slower recovery from illness and injury [7–14]. 

The collection of sociodemographic data is critical to understanding where equity gaps exist [15], but there remain challenges to collecting these data routinely. Barriers to collecting these data include patient discomfort in disclosing personal information and a lack of awareness of its intended purpose to improve health outcomes [16, 17]. For example, in a study conducted in Victoria, British Columbia, Canada, Indigenous participants did not feel safe disclosing their cultural background due to fear of discrimination [11]. Historically, there have been several examples of exploitation or mistreatment of marginalized groups, including racialized and Indigenous communities, that have led to mistrust of the healthcare system [18]. However, evidence also suggests that efforts by care providers to create supportive and safe environments can support patient willingness to share sensitive sociodemographic information such as race, ethnicity, sexual orientation, and gender identity [19, 20]. 

There is a paucity of literature that assesses patient perspectives on providing sociodemographic data in acute care settings, in particular, in the context of acute illness and with involvement of care providers outside of a primary care clinician. We therefore aimed to explore patient preferences and concerns around disclosing sociodemographic information in an acute care setting.

## Methods

We report our methods and findings in accordance with the Consolidated criteria for reporting qualitative studies (COREQ, Additional File 1) and the Guidance for Reporting Involvement of Patients and the Public 2 (GRIPP 2) checklist [21, 22] (Additional File 2).

### Research team and reflexivity

Research staff [YG, NP, JP, BB] from the Knowledge Translation Program at St. Michael’s Hospital-Unity Health Toronto in Toronto, Ontario conducted interviews. Interviewers were trained in qualitative methodology, held a bachelor’s or master’s level degree, and worked as research coordinators and assistants. Interviewers were of diverse racial backgrounds, three were women and one was a man.

Recognizing that interviewer identities, experiences and assumptions can influence the research process, the team engaged in reflexive discussions to examine how their positionalities might impact data collection [23]. Efforts were also made to reduce potential power imbalances between researchers and patients by having a direct care provider (i.e., physician, nurse, nurse practitioner) be the first point of contact for study recruitment. This approach aimed to foster trust and reduce the likelihood of pressure to participate due to perceived authority from the research team.

An interview guide with predefined questions and prompts was developed to ensure consistency in our data collection approach across interviewers. Patients eligible for participation were provided with a brief overview of the study by a direct care provider and/or a member of the study team (see Additional File 3). The interviewers did not hold any relationships with study participants. Additionally, the research team used a reflexive approach to interpret the study findings [23]. 

### Study design

#### Theoretical framework

We designed a qualitative study using the Framework Method [24] to explore patient perceptions on the routine collection of sociodemographic information in an acute care, urban hospital setting (e.g., through electronic medical records). The Framework Method is a systematic and flexible approach to the management and analysis of qualitative data. It involves seven stages: (1) transcription of recorded data; (2) familiarization with the data (e.g., by reading transcripts); (3) initial coding of a subsample of transcripts; (4) categorization of codes jointly decided on by members of the research team; (5) indexing (systematically applying the agreed upon framework); (6) charting data to summarize key content; and (7) interpretation (including theme development).

#### Patient engagement

Patient engagement in research can help improve study design, recruitment strategies, data quality and uptake [25–27]. We formed a study steering committee that included patients who participated in the development of the study protocol at project onset. Given COVID-19 restrictions, the patient steering committee was not involved in the conduct of the interviews.

#### Participant selection

Interviews took place between December 2021 and September 2023. The study setting included the General Internal Medicine (GIM) and Geriatrics Divisions at St. Michael’s Hospital-Unity Health Toronto in Toronto, Ontario (population of >6,000,000). St. Michael’s Hospital is a quaternary care hospital that is fully affiliated with the University of Toronto. It has over 450 inpatient beds and serves the city of Toronto’s downtown core.

Convenience sampling was used to recruit patients for interviews. Eligible participants were patients admitted to the GIM and Geriatrics Divisions at St. Michael’s Hospital, over the age of 18, able to speak English, and had the capacity to provide study consent. Physicians, residents and nurses helped to identify eligible participants. These clinicians were the first point of contact for participant recruitment. Research team personnel shadowed clinicians and once patients confirmed their interest in the study, a research team member collected informed consent. Recruitment and interviews were conducted in person at St. Michael’s Hospital by members of the research team [YG, NP, BB, JP].

Interviews were conducted in patients’ hospital rooms, which were sometimes shared with other patients. Curtains were drawn for privacy. The study background and consent terms were read to the participants prior to the interview start. Once interest in participation was confirmed, verbal informed consent was obtained, and an interview was conducted on the same or the following day.

### Data collection

Interviews lasted 10–30 min and were offered in English. No field notes were taken and there were no repeat interviews. The interviews were audio-recorded and transcribed verbatim using NVivo 12 Transcription [28]. Transcripts were not member checked but were reviewed for accuracy by the research team [NP, BB]. Identifying data were redacted prior to analysis. Participants received a $15 gift card for their participation in the study. All participants were presented with the option to complete a sociodemographic survey following the interview (see Additional File 4), which collected data on race; sex; gender; sexual orientation; education level; financial status; housing status; social supports; employment status; citizenship/immigration status; and whether the person experienced difficulty seeing, hearing, walking or climbing, remembering or with concentration, with self-care, or communicating.

### Analysis

Data were inductively analyzed for emergent themes using NVivo 12 [28]. Two analysts [NP, BB] independently reviewed a sample (10%) of the transcripts and developed open codes to generate a coding framework. The codebook was then systematically applied to the remainder of the transcripts; iterative revisions were made to the codebook as needed following a discussion between the analysts [24]. Two independent analysts then coded this sample of transcripts until a minimum kappa of 0.7 was reached [29]. The remaining transcripts were independently coded [BB] and summarized to present overarching themes [YG].

Sociodemographic questionnaire (Additional File 4) data were entered into Excel and analyzed using descriptive statistics; content analysis was used to analyze the open-ended questions by a single analyst [YG]. Any categories with fewer than 5 responses were collapsed or not reported in order to protect participant anonymity in accordance with Research Ethics Board policies.

### Ethics

This study was approved by Unity Health Toronto’s Research Ethics Board, REB study #20–300.

## Results

A total of 52 interviews were conducted between December 2021 and September 2023. Two additional patients were approached for recruitment but declined to participate due to concerns about confidentiality or having their data used for research purposes. Participants included 15 patients from the Geriatric Medicine inpatient unit and 37 patients from the GIM inpatient unit. From this sample, 21 participants (40%) completed the sociodemographic survey. The coders achieved a Cohen’s kappa of 0.84, indicating substantial agreement in coding.

### Survey: sociodemographic characteristics

Of the 21 participants (40%) who completed the sociodemographic survey, 57% (*n* = 12) were women and 43% were men (*n* = 9). The majority (62%,* n* = 13) of participants were White and 28% (*n* = 6) were racialized. Twelve participants (57%) were first generation immigrants. When asked about their functional and cognitive needs, 33% (*n* = 7) of participants reported difficulties with either walking or climbing stairs and an equal proportion reported difficulties with remembering or concentration. Additionally, 33% (*n* = 7) reported that they had difficulties making ends meet financially at the end of the month. Most (76%, *n* = 16) respondents reported that they rented or owned their home. Many participants (71%, *n* = 15) felt that they were able to rely on family/close friends for help. Table 1 outlines participant characteristics.

**Table 1:** Demographic characteristics

	Overall* (*n* = 21)	
	*n*	%
**Inpatient Unit**		
General Internal Medicine	11	52%
Geriatrics	10	48%
**Born in Canada**		
Yes	9	43%
No	12	57%
**Sex assigned at birth**		
Male	9	43%
Female	12	57%
**Current gender identity**		
Man	9	43%
Woman	12	57%
**Sexual orientation**		
Heterosexual	19	90%
Bisexual	---	---
Other	---	---
Prefer not to respond	---	---
Not reported	---	---
**Race/racial identity**		
Racialized	6	28%
White	13	62%
Prefer not to respond	---	---
**Highest level of education**		
Some college/university	7	33%
College/university degree and/or Postgraduate degree	9	43%
Prefer not to respond	5	24%
**Difficulty seeing**		
Yes	---	---
No	18	86%
Not reported	---	---
**Difficulty hearing**		
Yes	---	---
No	18	86%
Not reported	---	---
**Difficulty walking or climbing**		
Yes	7	33%
No	14	67%
Not reported	---	---
**Difficulty remembering or with concentration**		
Yes	7	33%
No	14	67%
Not reported	---	---
**Difficulty with communicating**		
Yes	---	---
No	20	95%
Not reported	---	---
**None of the above difficulties**		
Yes	7	33%
No	14	67%
Not reported	---	---
**Patient has access to family/close friends**		
Yes	19	90%
No	---	---
Not applicable/not reported	---	---
**Ability to rely on family/close friends**		
Yes	15	71%
No	---	---
Not applicable/not reported	---	---
**Current housing**		
Own home	7	33%
Rent	9	43%
Other	5	24%
**Living in social housing**,** subsidized housing**,** rent geared to income**		
Yes	---	---
No	7	33%
Not applicable	12	57%
**Time when patient was not able to afford rent/mortgage**		
Yes	---	---
No	17	81%
Not reported	---	---
**Employment in a casual**,** short-term or temporary position**		
Yes	---	---
No	19	90%
Not reported	---	---
**Large variation in pay month to month**		
Yes	---	---
No	5	24%
Not reported	8	38%
Not applicable	6	28%
**Difficulty making ends meet**		
Yes	7	33%
No	14	67%
Not reported	---	---
**Unable to afford prescription medication**		
Yes	---	---
No	19	90%
Not reported	---	---
**Fear of getting fired**		
Yes	---	---
No	7	33%
Not applicable	6	29%
Not reported	8	38%
**Missed Appointments**		
Yes	---	---
No	19	90%
Not reported	---	---
**Missed Payments**		
Yes	---	---
No	20	95%
Not reported	---	---

*cells that are < 5 have been suppressed to protect participant anonymity

### Preferences in providing sociodemographic information

Most participants had prior experiences with sharing their sociodemographic information in a healthcare setting. Of these individuals, the majority had positive experiences. We identified six key themes from the qualitative interviews, as presented below.

#### Patients would be willing to disclose sociodemographic data if they believed it would lead to better or more equitable care for themselves or for the health system

Most participants believed providing sociodemographic data in an acute care setting is important. Some believed these data were needed to facilitate equitable healthcare, while others were unable to explain why they felt it was important. Many participants felt that these data would help healthcare providers deliver a more tailored and informed approach to individual patient needs. A subset of participants were willing to share data if they believed it would directly impact the care provided to them during their hospital stay, as demonstrated below:

Yeah, so I think my question always is like as a patient, why are you asking? How is it going to impact me and my fellow patients in the future moving forward? – P06

Other participants valued the system-level benefits of collecting these data and believed these data provided a better understanding of population needs, as illustrated in the following participant quotes:

I think in a way it is actually [important]. If you use the information in there in a smart way. Because different ages, different races, you know, things that…if you are smart and analyzing the aspects, you can have a smart approach to a single individual. – P02

Predominantly studies have been on a particular…affluent demographic. And that doesn’t represent the entire world. So, it’s a very, very, very, very poor sample size. And therefore, the treatments that are built off of those aren’t necessarily tailored to individuals that are completely different, right? It’s more so just like trying to understand different groups of people because we’re trying to provide a care. Yeah, and we don’t know how to do it best. – P06

#### Reports of discomfort were greatest for the disclosure of income, followed by race

Most participants stated that they would be comfortable answering sociodemographic questions. More than half of participants were comfortable answering survey questions on race, income/income status, education, gender, sexual orientation, and immigration status. However, approximately one-third of participants expressed discomfort about completing at least one sociodemographic question; most often, these included questions on income, as demonstrated in the following quotations:

I don’t want to be judged in relation to [my] income. – P24

Income status should have no bearing on the hospital. You’re getting care from a hospital because obviously if you’re at the hospital, you need medical attention. What would giving your income status to hospital change for that...it just shouldn’t be needed. – P34

After income, discomfort reporting race was most commonly noted by patients. Notably, we observed differences in the reasoning behind why participants felt uncomfortable answering different demographic questions. For instance, participants who felt comfortable answering questions about race found the question offensive and unnecessary, whereas those who did not want to answer income questions expressed concerns about information security, as noted in the following participant quote,

Race is very sensitive. You have to be careful when asking about race. – P21

In addition to income and race, participants reported that they would not feel comfortable being asked about their sexual orientation, housing status and/or feelings of social isolation.

When participants voiced discomfort or hesitancy answering sociodemographic questions, it was largely due to a belief that this information was irrelevant to their care. Other common reasons included concerns about privacy, and concerns that disclosing this information may lead to differential treatment by healthcare providers due to biases, as described in the following quotations:

[I think the hospital knowing one’s demographic background would be a] bad thing. Wouldn’t it be? Because it’s like, it’s almost like discriminatory? – P34

I feel no need that skin colour or background should come into anything to do with the hospital. You’re sick. You know, you’re not checking the colour before you work on them. You’re bringing them in and you’re working on them. – P41

#### Most patients had no preference on who collects sociodemographic data

More than half of participants did not express a preference on which hospital staff member approaches them for sociodemographic data collection. Others preferred sociodemographic data to be collected by a doctor or nurse providing their care. Participants felt these were “safe” people to share these data with and that doctors and nurses would be more likely to use this information to help them, as shown in the following two quotations:

I think that nurses and doctors could be safe people to share information. Yeah. Also, because you are already sharing a lot of information, right? – P02

I think that [doctors] have a better understanding of why this information is being gathered. – P27

Conversely, some participants noted that a researcher would be a better choice to collect these data since they do not make decisions regarding patient care. In these cases, participants felt less worried about facing discrimination in their care as a result of information shared. Additionally, many participants expressed that the mannerisms and approach (i.e., a respectful, friendly demeanor) of the person collecting the data would impact their comfort, rather the person’s role.

Sometimes, like a nurse is like, really nice and like comes in like sits and then like, asks you questions and then it feels like nice and easy. But then sometimes they’re like doing a million things and like rushing and like, don’t care. – P19

With any [individual collecting the data] and as long as they are acting in a professional manner, I – you know, do those safety things to, you know, make sure it’s [the data are] not misrepresented, it’s not misused that I’d be comfortable with anything, anybody. – P35

#### Patients preferred being asked sociodemographic questions after being admitted to hospital

Most participants felt that these data should be collected once they were admitted to hospital rather than at intake, such as in the emergency department or at time of discharge. Typically, participants felt that once they were admitted, they would have more time to participate and would be more able (e.g., mental capacity, time) to answer questions, as represented in the following quotations:

I would say when you’re sitting down in your room all by yourself and just like staring at the wall, waiting for tests? Yeah, like today you came in at the right time. I just came back from an MRI. And I’m just, I’m moving upstairs, so it’s perfect. I got to talk to you. Yeah, I don’t have to rush out. I don’t have to, you know, I haven’t had a lot of things to deal with. – P35

Most people [when they] are being admitted, especially into emergency…the last thing they want to do is do a poll. They just want to get an I.V. and get into a bed. – P03

Those who felt that the collection of sociodemographic data would be best upon admission believed that having this information available to providers would help inform their care. Additionally, some participants expressed that the best timing would be whenever the patient had the most mental clarity.

#### Most patients preferred sociodemographic data to be collected verbally and in person, though others preferred a paper-based or online method

More participants preferred being asked sociodemographic questions verbally and believed it would provide them with an opportunity to ask questions, as described in the following quotations:

[Its good] if you have somebody else to explain because of course you [will] have questions. We feel more [comfortable] if we have somebody say “hi, welcome”…to come to talk. A lot of people [are] lonely [and] don’t have nobody here and no visitors, right? – P04

I like this [verbal] style because it’s easier for people to comprehend without having to go to read everything. – P30

I think verbal is better just because, like, when you’re tired, you don’t really want to write anything down. Yeah. So that would be my, my suggestion, I guess. Just keep it verbal. And online stuff, people usually don’t want to do those either. – P50

One participant noted that patients would appreciate the social interaction facilitated through verbal data collection while in hospital. Approximately one quarter of patients preferred completing sociodemographic questions online or did not have a preference; these participants typically cited convenience and comfort as the drivers of their preference. A minority of participants preferred to complete sociodemographic questions via a paper-based survey and believed a paper-based option would be more secure and easier to answer honestly.

#### Patients emphasized the importance of addressing concerns about data privacy, anonymization, use, and secure storage

Approximately one-quarter of participants expressed concerns about privacy when it came to sharing sociodemographic information. However, many of these individuals expressed that this concern would not prevent them from sharing their data. The most frequently cited concerns for providing sociodemographic data were hospital privacy breaches and a change in their treatment due to hospital staff’s biases, as demonstrated in the below quotes. Concerns noted included discrimination due to one’s demographic background, immigration status, and income.

There’s always risk. I mean, yeah, someone could get a hold of the information. Maybe. You never know. Especially now. – P50

I know what they do with that information. So that’s why I don’t give out that information. – P03

I think most people will be concerned with anonymity and their personal information being shared with someone they know, you know, or someone that may have some agenda. – P17

To alleviate concerns about privacy, the majority of participants wanted to know why these data were being collected and how the data would be used or shared. Many wanted to know that their data would be anonymized and securely stored, as shown in the following quotations:

I think as long as it’s like anonymous, I don’t think so. I don’t [think] there would be concerns. – P05

I think that the safety protocols put in place for our cyber information are adequate enough to restrict those who require the information. So as long as the safety protocols are followed and those who are given access are scrutinized over the conflict, OK. – P26

## Discussion

We assessed patient preferences in providing sociodemographic data in an acute care setting and identified six key themes. Patients interviewed expressed that they: (1) would be willing to disclose sociodemographic data if they believed it would lead to better or more equitable care for themselves or for the health system; (2) felt questions about income and race would create more discomfort than other sociodemographic questions; (3)had no preference about who collects sociodemographic data as long as their demeanor was friendly and respectful; (4) generally preferred being asked sociodemographic questions after being admitted to hospital; (5) preferred verbal, in person data collection; and (6) emphasized the importance of addressing concerns about data privacy, anonymization, use, and secure storage.

Similar to previous studies, our findings show that patients typically ascribe value to the importance of collecting sociodemographic data; however, perceptions on the purpose of the data collection and how it would be used varied [30–33]. Many participants assumed that the collection of sociodemographic data would be used to improve healthcare services, some did not understand the purpose of collecting these data, and others believed it would benefit the health system.

Most participants said that they would be more willing to share their data if they felt that it would improve healthcare for themselves or others. This is in line with previous literature suggesting that patients might be more comfortable reporting on sociodemographic characteristics when they have an understanding of how sociodemographic characteristics influence health, and how collecting these data can enhance healthcare [34, 35]. These findings demonstrate the importance of clarifying why data are being collected and how they will be used.

Participants who had concerns about sharing their data often cited a lack of understanding regarding the purpose of data collection and sometimes noted fears that noted fears that race-based discrimination could impact their care. This finding is consistent with previous studies conducted in acute care settings, where a lack of awareness regarding the purpose of data collection or lack of confidence about the usefulness of the data were identified as barriers to sociodemographic data collection [16, 36]. Transparently providing information on why and how data are being collected, used and stored may help to alleviate these concerns [16]. Evidence also suggests that factors such as a good relationship with the patient, or environments that are supportive of structurally marginalized populations (e.g., LGBTQ2SIA-friendly spaces) can support patients’ willingness to share sensitive sociodemographic information [19, 20]. 

While most participants stated that they were willing to provide their sociodemographic information, less than half agreed to complete a sociodemographic survey after the interview, further highlighting the challenges to collecting these data in hospital. Participants expressed discomfort around certain questions in particular, including questions on income and race. This is consistent with findings from another Toronto study where many survey respondents in a primary care setting chose not to disclose information about income and disability [19]. 

Our data also demonstrated differences in reasoning behind why participants did not want to answer certain questions. For instance, while fear of discrimination was most commonly noted as a concern for reporting data on race, those who reported on income were more likely to cite discomfort due to concerns about data security. Although the evidence is limited, some studies have reported similar trends. One study showed that most patients were not comfortable with a clinical decision-making algorithm rooted in race-based data, due to concerns that these data would lead to discrimination by healthcare providers [37]. 

Another study showed that participants were reluctant to report data on income due to concerns that that the questions did not allow them to represent their income or financial situation accurately [34]. This study also found that older adults were less likely to report on income [34]. This may help to explain the prevalence of hesitancy about reporting income in our sample, which was collected in Geriatrics and GIM units that typically serve an older adult population.

Overall, participants expressed discomfort reporting sociodemographic data due to fears of judgement, perceived lack of relevance, and data security concerns. A recent systematic review identified similar fears among patients about data sharing, irrespective of the type of data being shared [35]. 

Our findings also suggest that most patients did not have a preference about who was collecting the data, but highlighted the importance of a respectful and friendly demeanor from the person collecting the data. Studies confirm that the manner of data collection impacts patients’ likelihood of disclosing personal information [38]. Behaviours that help to build rapport include being non-judgmental, reassuring, compassionate, approachable, and empathic, listening respectfully, and engaging in shared decision-making [38]. 

Most participants in our study preferred in person, verbal collection of sociodemographic data, while others preferred a self-administered, paper-based or online method. Participants believed that their information would be more secure when provided verbally to a healthcare provider, and that self-administered surveys can create a greater burden on patients [16, 39]. However, there is also research indicating a preference for self-reporting personal information due to fear of judgment [40]. Self-administered surveys may facilitate individuals’ ability to respond honestly thereby minimizing social desirability bias [39, 41]. Thus, patients should be given the option of providing sociodemographic data verbally or via self-administered surveys.

Collecting sociodemographic data can facilitate healthcare through the better understanding of health disparities [42]. However, the collection of these data has the risk of causing harm, depending on how the data are collected and used [43]. Some potential harms include changes in care-seeking behaviour, concerns about data misuse, discomfort with or offense to questions, fear of discrimination, and fear of impact on quality of care [43]. To reduce patient harms, suggested best practices include: using statements that help to increase comfort and invite questions; providing a clear explanation of the need for sociodemographic data collection; transparency of use; and assurances about how privacy and confidentiality will be maintained [43]. 

Other recommendations based on a recent systematic review of sociodemographic data collection in emergency medicine settings are consistent with our findings and included: the provision of private spaces for information sharing; the use of categories that are not restrictive (e.g., options to decline disclosure); mandatory equity, diversity and inclusion training for staff; and avoiding repetitive disclosure [44]. Importantly, data collection strategies should be co-developed with patients, to ensure policies and procedures are aligned with patient values and are transparent, secure and equitable.

Our study contributes to the literature by providing insight into patient preferences for the modality and timing of sociodemographic data collection in an acute care setting. Additionally, we provide insights on patient preferences for data collection processes, which could facilitate implementation of routine data collection across hospitals in Canada and in similar contexts. 

There are several limitations to this study. Firstly, our findings are based on a convenience sample of patients admitted to the Geriatrics and GIM inpatient units of one urban, university-affiliated hospital and may not be generalizable to all patients in acute care settings. Further, data collection was conducted at bedside, which may have led to some social desirability bias in responses.

Additionally, it is important to consider the study sample’s demographic composition in the interpretation of our findings. Most participants who completed the sociodemographic survey identified as heterosexual, cisgender, and White. Most also reported at least some college/university education. As a result, it is possible that the perspectives of patients from other populations, including those who experience discrimination or marginalization, are underrepresented, which limits the generalizability of our findings. Due to a high rate of survey non-completion (60%), we were unable to disaggregate our themes to assess differences by sociodemographic characteristics. Although we did not formally explore why some participants declined survey participation, possible reasons may have included time constraints (e.g., waiting for discharge), lack of interest to continue with the data collection following a 20–30 min interview, or discomfort answering the questions. This gap presents a valuable area for future investigation.

There were also contextual and practical barriers that impacted recruitment and may have introduced selection bias. For example, hospital-wide pauses on non-COVID research projects during the study period led to delays and interruptions in study recruitment. After these pauses were lifted, COVID-19 outbreaks continued to impact multiple pauses in on-site research activities. Additionally, there was a risk of research fatigue among patients due to multiple studies recruiting from the same hospital units. Lastly, individuals who did not speak English were excluded from the study, which is an important limitation that may impact generalizability of findings, especially when considering health equity [31]. 

## Conclusions

Participants generally felt comfortable providing their sociodemographic information in acute care settings. However, the comfort level of some participants changed based on the approach, modality, and timing of data collection, suggesting that response rates may be enhanced by tailoring the administration of sociodemographic surveys to patient preferences. Perceived barriers to data collection included fears of stigma or discrimination by healthcare providers, a lack of knowledge about the purpose of the data collection, and concerns about data privacy. Respondents reported feeling most comfortable providing sociodemographic information through face-to-face, verbal disclosure. Additionally, participants emphasized the importance of a respectful approach of the interviewer, and largely felt that sociodemographic surveys should be administered once a patient has been admitted to hospital. Participants also highlighted the importance of clarifying the purpose of sociodemographic data collection and noted that privacy and confidentiality assurances are critical to ensuring patients feel comfortable sharing these data.

Future research should explore how to create accountability with patients and communities who share sociodemographic data with healthcare institutions, to ensure such data be used to narrow existing gaps in healthcare delivery and access.

## Supplementary Information

Below is the link to the electronic supplementary material.

Supplementary Material 1

Supplementary Material 2

Supplementary Material 3

Supplementary Material 4

## Data Availability

The datasets generated for this study are not publicly available to protect the privacy of participants and related REB confidentiality requirements. De-identified, themed data can be made upon request. To access the raw data analyzed in this study please contact Dr. Christine Fahim (christine.fahim@unityhealth.to).
