# Supplementary material for: Understanding patient preferences on providing sociodemographic information in an acute care setting: a qualitative study
Source: BMC Health Serv Res. 2025 Nov 22;25:1629. doi: 10.1186/s12913-025-13708-3 (PMC12750851; doi:10.1186/s12913-025-13708-3)
Supplement: Supplementary file 3 — Supplementary Material 3 [file 12913_2025_13708_MOESM3_ESM.docx]

**Appendix B**

**GEMINI Health Equity Questionnaire**

**Sociodemographic Information**

1. **If it could be arranged, would translation into another language be helpful at your next appointment?**
   - Yes
   - No

If yes, which language 🡪 _____________________

1. **Were you born in Canada?**
   - Yes
   - No

If no, what year did you arrive in Canada? ___________

1. **Do you identify as First Nations, Métis and/or Inuk/Inuit**?
   - Yes, First Nations
   - Yes, Métis
   - Yes, Inuk/Inuit
   - No
2. **In our society, people are often described by their race or racial background. These are not based in science, but our race may influence the way we are treated by individuals and institutions and this may affect our health. Which category(ies) best describes you? Check all that apply:**

- Black
- East Asian
- Indigenous (First Nations, Metis, Inuk/Inuit)
  - Do you identify as Two-Spirit? Yes/No
- Latino/Latina/Latinx
- Middle Eastern
- South Asian
- Southeast Asian
- White
- Another race category: ________________

1. **In general, do you experience any of the following due to a physical, mental, or emotional condition? *(select all that apply)***

- Difficulty seeing
- Difficulty hearing
- Difficulty walking or climbing
- Difficulty remembering or with concentration
- Difficulty with self-care
- Difficulty with communicating
- None of the above

1. **A) What was your sex assigned at birth? *(check one)***
   - Female
   - Male
   - Intersex
2. **B) What is your current gender identity? *(check one)***
   - Woman
   - Man
   - Transgender
   - Gender fluid or Gender nonbinary
   - Two-Spirit (Indigenous)
   - Another (Specify)__________
3. **Which best describes your sexual orientation?**
   - Heterosexual (“straight”, male/female relationships or two different binary genders)
   - Gay
   - Lesbian
   - Bisexual
   - Queer or pansexual
   - Two-Spirit (Indigenous)
   - Another (Specify)_____________

**Social needs**

1. **What is the highest level of education you have completed?**
   - Some grade school
   - Completed grade school (grade 1-8)
   - Some high school
   - Completed high school (grade 9-12)
   - Trades Certificate/Diploma
   - Some college/university
   - College/university degree
   - Postgraduate degree
   - No formal schooling
2. **Do you have difficulty making ends meet at the end of the month?**
   - Yes
   - No
3. **In the last 12 months, did you not fill a prescription or do anything to make a prescription last longer *because of the cost*?**
   - Yes
   - No
   - Not Applicable
4. **a) What is your current housing?**
   - Own home
   - Rent
   - Staying with friends or relatives because you have no alternative [couch surfing]
   - Shelter
   - On the street
   - Other (Specify)_______________

**b) If you rent 🡪 Is your current housing social housing, subsidized housing, or rent-geared-to-income?**

- - Yes
  - No
  - Not Applicable

**c) If own home/rent 🡪 During the last 12 months, was there a time when you were not able to pay the mortgage or rent on time?**

- - Yes
  - No
  - Not Applicable

1. **a) Do you feel you have family or close friends who you can open up to?**

- Yes
- No

**b) Are you able to rely on them if you need help (e.g., transportation, emotional or financial assistance)?**

- Yes
- No
- Not Applicable

1. **In the past 12 months, did you not attend an important appointment because of the cost of transportation?**

- Yes
- No
- Not Applicable

1. **In the past 12 months, did you miss making a payment on your electric, gas or other utilities bills *because of cost*?**

- Yes
- No

**16. a) Are you employed in a casual, short-term or temporary position?**

- Yes
- No

**b)** **Do you feel fearful that you could be fired if you raise employment concerns?**

- Yes
- No

**c) Does your pay vary a lot from month to month?**

- Yes
- No
