# Supplementary material for: Understanding patient preferences on providing sociodemographic information in an acute care setting: a qualitative study
Source: BMC Health Serv Res. 2025 Nov 22;25:1629. doi: 10.1186/s12913-025-13708-3 (PMC12750851; doi:10.1186/s12913-025-13708-3)
Supplement: Supplementary file 4 — Supplementary Material 4 [file 12913_2025_13708_MOESM4_ESM.docx]

**Appendix A**

**Interview Guide**

Please keep in mind that during this interview, I would like to know what your *preferences* are when being asked questions about your personal information (like your age, gender, racial/ethnic background, or income), but I am not asking you to *provide* your personal information. For example, I will ask you what you prefer when being asked about your gender, but not what your gender is.

***Interview***

Opening question:

1. I would like to begin by asking if you’ve had any experiences in providing personal demographic information (for example age, gender, race/ethnic background, income) at a hospital before?

- [*If yes*] *please describe. What was the purpose of being asked these questions? What was the role of the individual asking you questions?*
- [*If no*] *have you been asked these kinds of questions in non-hospital settings? If yes, please describe. What was the purpose of being asked these questions? What was the role of the individual asking you questions?*

***Research question #1: What are patients’ perceptions of the importance of providing their sociodemographic information to their healthcare provider?***

1. Do you think it is important for the hospital to know information about your background (e.g., your race/ethnic background, income, housing status)? *Why or why not?*
2. Do you think there are any risks having to do with the hospital having this information (for example, how this information will be used or managed by the hospital)
   - *[If yes] What are these concerns? What would help alleviate those concerns?*

***Research question #2: What sociodemographic questions would patients be willing to answer and have included in their health records*?**

1. I’m going to read out a list of personal information you might be asked about in a hospital setting. Please let me know if you would be comfortable answering these questions, or if there are any that you would be uncomfortable answering.
   Please say “yes” if so and “no” if you would not feel comfortable. Would you be comfortable answering a question about your:
   - Race
   - Ethnic background
   - Language
   - Income
   - Education
   - Gender
   - Sex *(If patient is confused between gender and sex: sex is male, female, intersex, etc. and is assigned at birth, gender is man, woman, non-binary, etc. and is a personal identity)*
   - Sexual orientation
   - Housing status
   - Income status
   - Immigration status
   - Social isolation

*Probe for why for each identifier and/or if there any cultural values, preferences that should be considered when being asked to provide this information.*

*If patient starts to answer the question with their own sociodemographic information (e.g. race, ethnic background, etc.) redirect the question to* ***how comfortable they are with being asked about that information, not what their own information is – ask them to respond “yes” if they feel comfortable providing this information and “no” if they do not.*** *Providing answers to the sociodemographic question in the form of providing their own information is not an indication of comfort.*

1. Can you provide any suggestions on how we could ask about race, income, education, etc.? (*Can refer to GEMINI Health Equity Questionnaire*)

***Research question #3: How do patients describe their preferred method of providing and storing their sociodemographic information? (how, when, who, where)***

1. In the future, how would you prefer to provide your demographic information: paper, online, or verbally?
2. Which hospital staff members (administrator, researcher, nurse, physician) would you feel comfortable giving your personal demographic information to?
3. What do you think is the best time to provide this information? (e.g., upon arrival, during the admission process, in the ER, in the room after admission)
4. What kind of information would you want to know before being asked questions about your personal information?

*[If the patient completed the GEMINI Health Equity Questionnaire*]

Were you able to complete the [*GEMINI Health Equity Questionnaire*]?

If yes,

1. Did you experience any discomfort in completing the questionnaire/answering any of the questions? Any other concerns (confidentiality, question clarity)?
2. Do you have any suggestions for improving the process of collecting sociodemographic information in a hospital setting?

We’ve come to the end of our interview, is there anything else you would like to add?
